# Characterizing the molecular composition and diagnostic potential of *Mycobacterium tuberculosis* urinary cell-free DNA using next-generation sequencing

**DOI:** 10.1016/j.ijid.2021.09.042

**Published:** 2021-11

**Authors:** Amy Oreskovic, Adam Waalkes, Elizabeth A. Holmes, Christopher A. Rosenthal, Douglas P.K. Wilson, Adrienne E. Shapiro, Paul K. Drain, Barry R. Lutz, Stephen J. Salipante

**Affiliations:** a Department of Bioengineering, University of Washington, Seattle, Washington, USA; b Department of Laboratory Medicine and Pathology, University of Washington, Seattle, Washington, USA; c Umkhuseli Innovation and Research Management, Pietermaritzburg, South Africa; d Edendale Hospital, University of KwaZulu-Natal, Pietermaritzburg, South Africa; e Department of Medicine, University of Washington, Seattle, Washington, USA; f Department of Global Health, University of Washington, Seattle, Washington, USA; g Brotman Baty Institute for Precision Medicine, Seattle, Washington, USA

**Keywords:** *Mycobacterium tuberculosis*, DNA sequencing, Cell-free DNA, Transrenal DNA, Urine, Diagnostics, Molecular diagnosis

## Abstract

**Background:**

Urine cell-free DNA (cfDNA) is an attractive target for diagnosing pulmonary *Mycobacterium tuberculosis* (MTB) infection, but has not been thoroughly characterized as a biomarker.

**Methods:**

This study was performed to investigate the size and composition of urine cfDNA from tuberculosis (TB) patients with minimal bias using next-generation sequencing (NGS). A combination of DNA extraction and single-stranded sequence library preparation methods demonstrated to recover short, highly degraded cfDNA fragments was employed. Urine cfDNA from 10 HIV-positive patients with pulmonary TB and two MTB-negative controls was examined.

**Results:**

MTB-derived cfDNA was identifiable by NGS from all MTB-positive patients and was absent from negative controls. MTB cfDNA was significantly shorter than human cfDNA, with median fragment lengths of ≤19–52 bp and 42–92 bp, respectively. MTB cfDNA abundance increased exponentially with decreased fragment length, having a peak fragment length of ≤19 bp in most samples. In addition, we identified a larger fraction of short human genomic cfDNA, ranging from 29 to 53 bp, than previously reported. Urine cfDNA fragments spanned the MTB genome with relative uniformity, but nucleic acids derived from multicopy elements were proportionately over-represented.

**Conclusions:**

TB urine cfDNA is a potentially powerful biomarker but is highly fragmented, necessitating special procedures to maximize its recovery and detection.

## Highlights

• A robust methodology to study *Mycobacterium tuberculosis* (MTB)-derived cell-free DNA (cfDNA) from patient urine was developed.
• The MTB-derived cfDNA length was found to be shorter than previously appreciated (peak ≤19 bp).
• MTB cfDNA from multicopy elements were proportionately over-represented.
• Special methods are needed to maximize the recovery and detection of urinary MTB cfDNA.

## 1 Introduction

There is a critical need for diagnostics for pulmonary *Mycobacterium tuberculosis* (MTB) infection that do not require sputum collection, which is difficult in many patients. Even when sputum is available, existing assays have reduced sensitivity for diagnosing paucibacillary, HIV-associated, pediatric, and extrapulmonary tuberculosis (TB) (Detjen et al., 2015; Horne et al., 2019; Kohli et al., 2018). Transrenal urine cell-free DNA (cfDNA) is a promising, easy-to-collect biomarker for MTB with the potential to diagnose patients from these groups (Cannas et al., 2008; Green et al., 2009; Labugger et al., 2016; Leticia Fernández-Carballo et al., 2019; Oreskovic et al., 2021; Patel et al., 2018), but has not yet been extensively characterized or validated as an analyte.

In particular, the fragment length distribution of transrenal microbial cfDNA, including MTB cfDNA, could have a strong influence on the recovery and detection of this material, but is not well understood. Recent work identified short MTB urine cfDNA fragments by next-generation sequencing (NGS) (19–44 bp), but minimally characterized the size distribution of those fragments (Sinkov et al., 2019). Moreover, conventional methods for DNA extraction and NGS likely underestimate the proportion of short cfDNA molecules present, because they have poor retention of degraded DNA fragments (Oreskovic et al., 2019; Shekhtman et al., 2009). Consequently, the molecular properties of MTB urinary cfDNA, and practically, the methods best suited to purify and identify those fragments for diagnostic purposes, remain incompletely explored.

The aim of this study was to better characterize the fragment length distribution of MTB-derived urine cfDNA using NGS and to identify any potentially over-represented sequences suitable for targeting in diagnostic assays. Single-stranded NGS library preparation was utilized (Gansauge and Meyer, 2013; Troll et al., 2019), which improves the yield of short cfDNA fragments (<100 bp) and recovers highly degraded forms of cfDNA, including microbial cfDNA, which is less protected from nuclease digestion than human genomic material (Burnham et al., 2018, 2016). In addition, a DNA extraction method with the highest affinity for short, urine cfDNA was used (Oreskovic et al., 2019; Shekhtman et al., 2009). It was theorized that this combination of methods would minimize biases relating to fragment length and enable more accurate NGS characterization of MTB urine cfDNA.

## 2 Methods

### 2.1 Participant enrollment and urine collection

Participants were enrolled at Edendale Hospital in Pietermaritzburg, South Africa between October 2019 and February 2021. Adults (≥16 years old) with MTB-positive admission sputum and individuals with HIV were recruited and screened for sputum MTB positivity using Xpert MTB/RIF Ultra (Cepheid) testing. Patients with >24 hours of anti-TB treatment were excluded. All participants provided written informed consent.

Participants were provided with a sterile specimen cup to collect a morning urine sample. Urine (50–200 ml) specimens were immediately mixed in 10-ml aliquots with ethylenediaminetetraacetic acid (EDTA) (final concentration 25 mM) and Tris-HCl pH 7.5 (final concentration 10 mM), and stored in DNA LoBind tubes (Eppendorf) at −80°C.

### 2.2 Clinical data, sputum testing, and urine lipoarabinomannan (LAM) testing

The following clinical data were collected: sex, TB symptoms, TB treatment duration, HIV test result, and CD4^+^ cell count. Participant sputum was submitted to the South African National Health Laboratory System (NHLS) for Xpert MTB/RIF Ultra testing and confirmatory mycobacterial culture. Mycobacterial culture was performed for up to 42 days at the NHLS Provincial TB Reference Laboratory using Middlebrook 7H11 solid agar medium and the liquid BACTEC mycobacterial growth indicator tube (MGIT) 960 system (BD). Culture plates were read at 3 and 6 weeks, and MTB was identified using niacin and nitrate testing. Urine (60 µl) was tested using the Alere Determine TB LAM Ag test (Abbott Laboratories). Participants were categorized as TB-positive if either Xpert MTB/RIF Ultra or mycobacterial culture was positive, or TB-negative if neither was positive and no clinical TB diagnosis was established within 2 months of enrollment.

### 2.3 cfDNA extraction using Q Sepharose anion exchange resin

Urine was thawed at 37°C and centrifuged for 5 min at 8000 *g*. Supernatant was transferred to new 15-ml DNA LoBind tubes and DNA was extracted using Q Sepharose as described previously (Oreskovic et al., 2019), with some modifications. Specifically, the spin speed of the QIAquick column wash was increased to 8000 *g*, the elution volume was reduced to 50 µl, and the PCR template was reduced to 2 µl. These modifications had no effect on cfDNA yield or the recovery of a 50-bp positive control sequence (not shown).

### 2.4 Quantification of cfDNA

Total cfDNA was measured using the Qubit dsDNA HS Assay Kit (Thermo Fisher Scientific). MTB complex-specific cfDNA was measured by qPCR of IS6110, as described elsewhere (Oreskovic et al., 2021).

### 2.5 Next-generation sequencing

Sequencing libraries were prepared with 1–45 ng purified cfDNA using the SRSLY method, as described elsewhere (Troll et al., 2019), or the commercially available formulation, SRSLY PicoPlus kit (Claret Biosciences), with some modifications. Specifically, to retain low molecular weight cfDNA fragments, the Monarch Genomic DNA Purification Kit (NEB) was used for library purification after phosphorylation/ligation, final library purification was performed using 1.8 × volumes AMPure XP beads (Beckman Coulter), and size selection was not performed. Two to 11 replicates were generated per specimen. Sequencing utilized a NextSeq 500 (Illumina) with 150 bp paired-end chemistries.

### 2.6 Data analysis

Sequence reads from replicates were combined prior to analysis. Reads were trimmed using fastq-mfc of ea-utils-1.1.2.779 (Aronesty, 2013), with a minimum retained length of 15 bp. The taxonomic composition of reads was cataloged using kraken2 (Wood et al., 2019). Read pairs classified as human (down-sampled to 200 000 reads) or as *Mycobacterium* genus were isolated. Read mapping was performed against the human genome (hg38) or the MTB H37Rv genome (GenBank accession number **AL123456.3**) using BWA-MEM (v0.7.12) (Li, 2013) with default parameters. Reads with mapping quality of ≥5 were retained. This approach identifies fragments ≥19 bp in length, the default minimum seed length required by BWA-MEM. cfDNA fragment length distributions were determined using deepTools (Ramírez et al., 2016), with the “distanceBetweenBins” flag set to 100.

For studies of multicopy elements IS6110 and IS1081, reads were mapped directly to those sequences (GenBank accession numbers **X17348.1** and **X61270.1**, respectively) using BWA-MEM as above, and read counts quantified. The statistical analysis was conducted using GraphPad Prism v8.1.2, with a significance level of 0.05.

### 2.7 Data availability

Reads mapping to MTB are available from the NCBI Sequence Read Archive (SRA) under accession number **PRJNA725220**.

## 3 Results

### 3.1 Q Sepharose DNA extraction and cfDNA quantification

cfDNA was extracted from the urine of 29 TB-positive and five TB-negative participants by Q Sepharose. MTB-specific cfDNA was detected using IS6110 qPCR in 14/29 (48.3%) samples from TB-positive participants and 0/5 (0%) samples from TB-negative participants (Table 1).

**Table 1:** Concentrations of total and MTB-derived urine cfDNA detected after Q Sepharose extraction^a^

		Median (IQR)	Range
Total cfDNA concentration^b^	Eluate (ng/µl)	5.1 (2.7–11.9)	1.1–85.6
	Urine (ng/ml)	25.5 (13.3–59.3)	5.4–428
Estimated MTB cfDNA concentration^c^	Eluate (copies/µl)	5.2 (0.8–6.5)	0.1–792
	Urine (copies/ml)	26 (4.0–32.4)	0.6–3958

cfDNA, cell-free DNA; IQR, interquartile range; MTB, *Mycobacterium tuberculosis*.

a The detected concentrations of total and MTB-specific cfDNA in each sample selected for sequencing are given in Supplementary Material Table S1.

b Measured using the Qubit HS dsDNA kit.

c Measured by 40 bp qPCR targeting the variable copy number insertion sequence IS6110.

### 3.2 Urine cfDNA sequencing

Nine TB-positive samples with the highest concentrations of MTB-specific cfDNA, one TB-positive sample without qPCR detectable MTB cfDNA, and two TB-negative samples were selected for single-stranded library preparation and sequencing (Supplementary Material Table S1). All participants were HIV-positive with a median CD4 count of 141 cells/mm^3^ (interquartile range 59–516 cells/mm^3^). Participants were 42% female and 58% male. Fifty percent of TB-positive participants had a positive urine LAM.

Thirty million to 113 million sequence reads were generated per specimen, resulting in 24–99 million reads after initial quality filtering to remove self-ligated adaptor sequences (Supplementary Material Table S2). Library complexity, the measured proportion of unique sequence fragments sequenced per library, was high for all cases, ranging from 95.2% to 100%.

### 3.3 Urine cfDNA taxonomic composition

The taxonomic composition of cfDNA reads was characterized using metagenomic analysis (Wood et al., 2019) (Supplementary Material Table S2, data available from the authors on request). For all cases, the majority of quality-filtered sequence reads (84.5–99.2%) corresponded to human nucleic acid, while the next most abundant taxa were attributable to microorganisms comprising the normal skin or genitourinary microbiota, primarily species within Actinobacteria, Proteobacteria, and Bacteroidetes (Pearce et al., 2014). The proportion of reads originating from bacteria of any kind averaged 1.71% per case (range 0.57–3.69%), with no difference in bacterial sequence load between TB-positive and TB-negative study participants (*P* = 0.71, two-tailed *t*-test). The remaining reads were distributed among higher level taxonomic classifications, viral and phage sequences, and unclassified reads.

Sequences putatively classified as MTB or human were mapped against their respective reference genomes to confirm identity. Following this quality control step, MTB-derived reads were identified in all specimens from TB-positive participants (10/10), including the patient lacking qPCR-detectable MTB (Supplementary Material Table S2). Significantly, no reads mapping to the MTB genome were identified from either of the TB-negative individuals (0/2). An average of 2332 reads originated from the MTB genome in TB-positive patients (range 4–19 547 reads), corresponding to 0.00001–0.0201% of total reads.

### 3.4 Human and MTB urine cfDNA fragment length distributions

**Figure 1:**
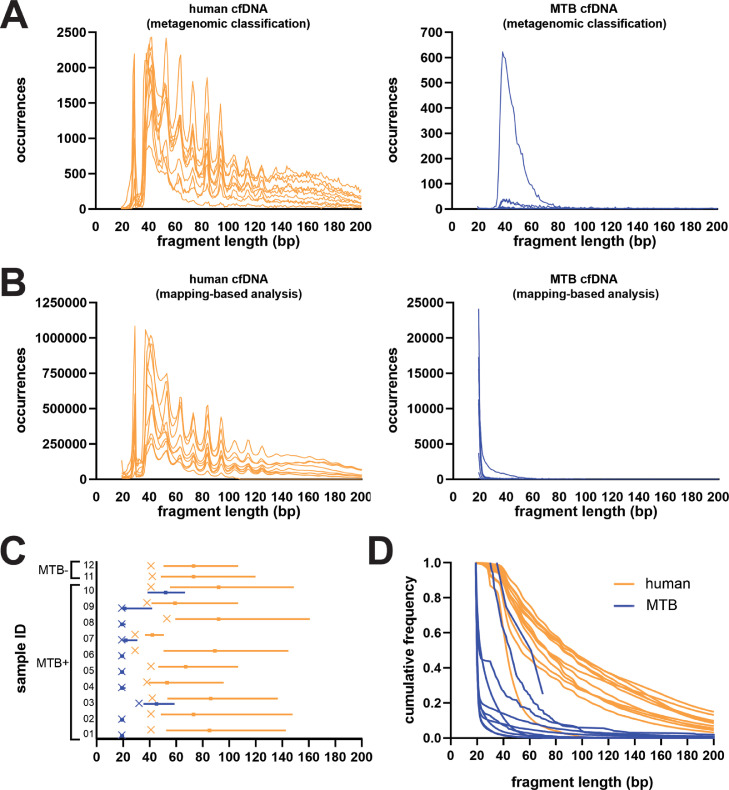
MTB urine cfDNA is significantly shorter than human urine cfDNA. (A) Fragment length distributions of urine cfDNA in each sample classified as human (orange, *n* = 12) and MTB (blue, *n* = 10) by metagenomic analysis techniques. (B) Fragment length distributions of urine cfDNA in each sample mapped to the human genome (orange, *n* = 12) and MTB genome (blue, *n* = 12). Individual plots for each sample are given in Supplementary Material Figure S1 and Supplementary Material Figure S2. (C) Characterization of fragment length for cfDNA mapped to the MTB genome (blue, *n* = 10) and human genome (orange, *n* = 12) in each sample. Bars indicate the median fragment length and interquartile range (IQR). ‘x’ indicates the mode fragment length. No mode length is shown for sample 10 because it was multimodal with a low number of reads mapped to MTB. The median, IQR, and mode fragment length for each individual sample are given in Supplementary Material Table S1. (D) Cumulative frequency of MTB (blue, *n* = 10) and human genomic (orange, *n* = 12) cfDNA by fragment length in each sample. (MTB, *Mycobacterium tuberculosis*; cfDNA, cell-free DNA.)

Next, sequencing data were used to explore the length of cfDNA fragments derived from human and MTB genomes (Figure 1).

Human cfDNA showed a relatively broad distribution of fragment lengths, with abundance inversely proportional to fragment length (Figure 1A, left; Supplementary Material Table S1). A periodicity in abundance occurred at approximately 10-bp intervals, as expected based on nucleosome length and previous cfDNA sequencing studies (Burnham et al., 2018; Cheng et al., 2017; Markus et al., 2021; Snyder et al., 2016; Tsui et al., 2012). The most abundant fragment length for human urine cfDNA ranged from 28 to 53 bp across samples. The median fragment lengths for human urine cfDNA were 45–97 bp.

The abundance of MTB urine cfDNA similarly increased with decreasing fragment length. However, in contrast to human cfDNA, MTB-derived cfDNA displayed no periodicity and showed a left-shifted distribution (Figure 1A, right; Supplementary Material Table S1). In samples having enough reads to determine the most abundant MTB cfDNA fragment length, this value ranged from 38 to 43 bp. Median fragment lengths for MTB-derived urine cfDNA were 39–97 bp and were significantly shorter than those of human cfDNA (*P* = 0.02, Wilcoxon matched pairs test).

Although this analysis recovers MTB-derived cfDNA with high specificity, a drawback is that it preferentially identifies longer sequences, which have a correspondingly higher probability of containing sequence motifs that uniquely identify them as MTB. Shorter fragments that legitimately derive from the MTB genome are more likely to share significant similarity with other species by homology or chance, and will be excluded.

To provide an analysis that is less biased with respect to sequence length, all sequence reads were next aligned to the MTB and human reference genomes, and those that could be successfully mapped were retained for analysis. A comparatively greater number (average 22 545, range 4–78 240) and proportion (average 0.027%, range 0.00001–0.081%) of reads matching the MTB genome were recovered from TB-positive patients (Supplementary Material Table S2). A small number of reads from TB-negative participants also mapped to the MTB genome, suggesting minor, artifactual contributions of cfDNA from other organisms that have been mapped to the MTB genome. Nevertheless, the proportions of MTB-mapped reads from negative patients (0.0000318% and 0.000178% of reads, corresponding to read counts of 9 and 42, respectively) were three orders of magnitude less than the average for TB-positive patients, despite the two groups having comparable proportions of total bacterial cfDNA by metagenomic analysis. Moreover, no correlation was found between the proportion of reads mapping to the MTB genome and the proportion of total bacterial reads by metagenomic analysis (Pearson correlation coefficient *r* = 0.0708, *P* = 0.83), but a significant positive correlation was observed between the proportion of reads mapping to the MTB genome and the proportion that were unambiguously classified as MTB by our high specificity approach (Pearson correlation coefficient *r* = 0.6727, *P* = 0.017). Taken together, these findings indicate that the contributions of non-MTB organisms to the analysis are minor, and that the reads being mapped to the MTB genome are mainly attributable to MTB-derived cfDNA.

While the length distribution of human reads by this approach was consistent with our earlier results (Figure 1B, left; Supplementary Material Table S2), with a peak cfDNA fragment length of 29–53 bp, cfDNA fragments mapping to the MTB genome were substantially shorter than previously indicated (Figure 1B, right). The abundance of MTB urine cfDNA increased exponentially with decreasing fragment size (Figure 1B, right) and showed a peak fragment length of ≤19 bp, the minimum size detectable by our analysis, in most samples (8/10) (Figure 1C). The median fragment lengths for MTB-derived urine cfDNA (≤19–52 bp) remained significantly shorter than for human urine cfDNA (42–92 bp) (*P* = 0.002, Wilcoxon matched pairs test, Figure 1C).

Mapped reads were used for subsequent analyses.

### 3.5 Distribution of MTB-derived reads across the genome

**Figure 2:**
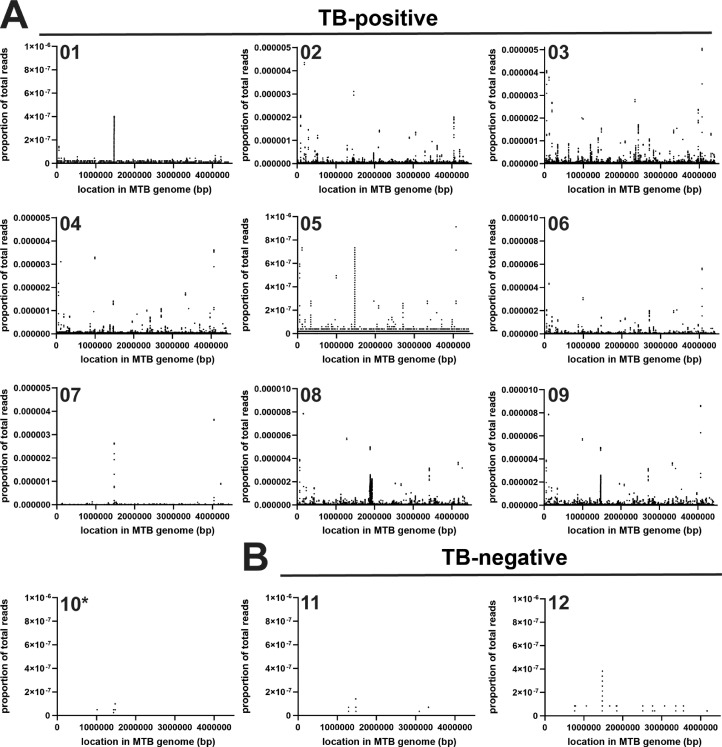
Coverage of the MTB genome in urine cfDNA. (A) Density of reads mapped to the MTB genome in 10 samples from TB-positive participants. *Sample 10 had no MTB cfDNA detectable by IS6110 qPCR, but MTB-specific cfDNA was detectable by sequencing and confirmed by metagenomic classification analysis (kraken2). (B) Density of reads mapped to the MTB genome in two samples from TB-negative participants. (MTB, *Mycobacterium tuberculosis*; cfDNA, cell-free DNA.)

In TB-positive participants, cfDNA reads mapping to the MTB reference genome showed low but relatively uniform coverage across the genome (Figure 2A). Notably, for most samples, the rRNA gene locus (positions 1 471 846–1 477 013 bp) evidenced increased read coverage relative to the rest of the MTB genome, despite there being a single copy of this locus carried by MTB (Stoddard et al., 2015). As rRNA encodes an essential gene that is highly conserved across bacterial taxa (Clarridge, 2004), these data suggest that short reads from other organisms present in patient specimens may infrequently map to the MTB genome at specific sequence contexts.

### 3.6 Multicopy elements in the MTB genome as diagnostic targets for urine cfDNA

Species-specific multicopy elements are attractive targets for diagnostic testing because they provide both specificity and inherent signal amplification. To evaluate two known multicopy elements as potential urine cfDNA diagnostic targets, the relative abundance of reads mapping to two insertion sequences (IS6110 and IS1081) present in the MTB genome were analyzed (Collins, 1991; Thierry et al., 1990).

Fragments derived from IS6110 and IS1081 cfDNA were detected by NGS in 6/9 and 5/9 specimens having MTB cfDNA detectable qPCR, respectively (Table 2). In cases where these sequences were identified they were found with greater abundance than reads from other regions of the MTB genome. The average fold over-representation of IS6110 relative to the average sequencing depth for the remainder of the MTB genome was 8.1 (range 1.3–17.1), whereas that observed for IS1081 was 4.6 (range 1.7–8.5). These values correspond roughly to the expected count of each element per genome: IS6110 is present at a variable copy number of 0–25 across MTB complex strains (del Carmen Menéndez et al., 2012), while IS1081 is more stable at 5–6 copies (van Soolingen et al., 1992). It was not possible to examine reads mapping to a third multicopy element used in TB studies, the direct repeat (DR, 14–63 copies) region (Beggs et al., 1996), given the repetitive nature of the element and the short length of its constituent repeat sequence (36 bp), which prevented reliable read mapping.

**Table 2:** Relative abundance of multicopy elements IS6110 and IS1081 in urine cfDNA

Sample ID	TB status	cfDNA status (IS6110 qPCR)	IS6110 fold over-representation^a^	IS1081 fold over-representation^a^
01	Positive	Positive	0	0
02	Positive	Positive	0	0
03	Positive	Positive	17.0	2.52
04	Positive	Positive	1.27	0
05	Positive	Positive	3.30	6.78
06	Positive	Positive	3.68	3.62
07	Positive	Positive	17.1	8.48
08	Positive	Positive	6.34	1.72
09	Positive	Positive	0	0
10	Positive	Negative	0	0
11	Negative	Negative	0	0
12	Negative	Negative	0	0

cfDNA, cell-free DNA; IQR, interquartile range; MTB, *Mycobacterium tuberculosis*.

a Measured as the average sequencing depth in the target region normalized to the average sequencing depth across the remainder of the MTB genome.

## 4 Discussion

This study presents an in-depth, minimally biased characterization of MTB urine cfDNA using NGS, with a focus on defining its properties relevant to molecular diagnosis. To most comprehensively characterize the full range of cfDNA fragments, DNA extraction and sequencing library preparation methods were selected specifically for their demonstrated effectiveness for short DNA fragments. We have previously shown in comparisons of urine cfDNA purification methods that Q Sepharose extraction, which pre-concentrates urine cfDNA using anion exchange resin prior to desalting on a silica spin column (Shekhtman et al., 2009), has high recovery (>70%) of DNA at least 40 bp in length (Oreskovic et al., 2019). Recovery by that method is reduced to <10%, but is still measurable, for DNA as short as 25 bp in length (Oreskovic et al., 2019). Similarly, single-stranded NGS library preparation (Gansauge and Meyer, 2013; Troll et al., 2019) has been shown to improve the recovery of <100 bp cfDNA, with a lower reported limit of 40–60 bp (Burnham et al., 2016). In this application, we have further extended the lower range of detection for this approach by retaining all library fragments generated, which increased the sensitivity for low molecular weight DNA fragments at the expense of sequencing an increased proportion of synthetic, noncontributory fragments resulting from self-ligated sequencing adaptor molecules (measured at 9–20% of total reads generated per specimen).

Previous NGS studies characterizing the fragment length distribution of human genomic cfDNA have reported peak fragment lengths ranging from approximately 50 to 100 bp (Burnham et al., 2018; Cheng et al., 2017; Markus et al., 2021). In contrast, the present study demonstrably improved the recovery of short cfDNA fragments and revealed a previously undetectable fraction of human genomic cfDNA in urine, with the most abundant fragment length ranging from 29 to 53 bp among the samples examined. The differences between our methodology and protocols employed previously were most noticeable for the shortest fragments, with representation of <50 bp fragments in the present study dramatically increased relative to earlier work that did not use single-stranded library preparation methods (Cheng et al., 2017; Markus et al., 2021; Tsui et al., 2012) or that used single-stranded library preparation in conjunction with a DNA extraction method less able to recover short DNA fragments (Qiagen Circulating Nucleic Acid Kit) (Burnham et al., 2018).

It was found that MTB cfDNA in urine is extensively fragmented, significantly more so than human genomic cfDNA, having a peak size of ≤19 bp. To maximize the clinical sensitivity of MTB urine cfDNA assays, both sample preparation and amplification methods having high efficiency for very short fragments will consequently be needed. Specialized DNA purification procedures are necessary to recover fragments in the size range containing MTB cfDNA (Oreskovic et al., 2019; Shekhtman et al., 2009), and must be employed to optimize diagnostic yields. Separately, decreasing the minimum detectable target length improves the detection sensitivity for fragmented cfDNA (Chan et al., 2008; Melkonyan et al., 2008; Shekhtman et al., 2009) and has been a priority during the recent development of MTB urine cfDNA assays (Cannas et al., 2008; Labugger et al., 2016; Oreskovic et al., 2021; Patel et al., 2018). Previously-reported MTB urine cfDNA assays targeted, at the shortest, amplicons of 38–40 bp (Labugger et al., 2016; Oreskovic et al., 2021; Patel et al., 2018). Decreasing the PCR amplicon length from 49 bp to 39 bp resulted in a greater than 10-fold increase in detected MTB cfDNA (Melkonyan et al., 2008). Until now, the extent to which further decreases in target length may improve sensitivity has been unclear. Our results suggest that even small, incremental decreases in target length may have a disproportionate impact on the detection of MTB urine cfDNA, which increases in abundance exponentially as fragment size decreases. Ultrashort PCR using a stem-loop primer may be an attractive strategy for amplification of fragments too short for conventional PCR (Shekhtman et al., 2009). Alternatively, recent work by our group demonstrated that sequence-specific purification improves the recovery of short cfDNA relative to conventional silica-based extraction and increases the clinical sensitivity of TB diagnosis from urine cfDNA (Oreskovic et al., 2021; Oreskovic and Lutz, 2021). Moreover, the present results, in concert with those of a previous study (Sinkov et al., 2019), suggest that targeting multicopy genomic elements (e.g., IS6110, IS1081) is likely a more promising strategy than the identification of highly represented cfDNA targets de novo.

This study has several limitations. First, cfDNA only from people living with HIV was sequenced. Although the detection sensitivity for MTB urine cfDNA is similar in HIV-positive and HIV-negative participants (Oreskovic et al., 2021; Patel et al., 2018), it remains unclear whether there are differences in cfDNA fragmentation patterns across these two populations. Second, owing to the requirements for high sequencing depths and attendant sequencing costs, the number of specimens analyzed in this study are necessarily limited. Third, despite the improvements in short cfDNA fragment recovery using a combination of Q Sepharose DNA extraction and single-stranded library preparation, the methods are unable to reliably interrogate the shortest cfDNA fragments. It is expected that the efficiency of fragment recovery begins to decrease below 40 bp (Oreskovic et al., 2019), and due to the nature of sequence read mapping algorithms, it is not possible to reliably map the origin of sequence reads below a specified seed length (here, 19 bp). Moreover, the shorter the fragment length, the less probable it is that the resultant read will map confidently to its target (Li and Freudenberg, 2014). Considering these limitations, the true frequency of cfDNA <40 bp in length, whether originating from human or MTB, is likely even greater than registered by our analysis. The fragment length distribution of cfDNA should be interpreted with this in mind. Fourth, many of the cfDNA molecules recoverable by our methods are so short that they cannot be uniquely classified as belonging to MTB. As a consequence, it is not possible to demonstrate directly that all of the smallest read fragments that were mapped to MTB derive from that organism, although accessory evidence is consistent with that conclusion.

In summary, accurate characterization of urine cfDNA using NGS provides a critical insight into its validation as a biomarker for MTB. The study findings, particularly the discovery that MTB cfDNA is substantially shorter than human genomic cfDNA, will help inform the development of improved assays for TB diagnosis from urine cfDNA. The large potential sensitivity benefit to be gained by targeting <40 bp MTB cfDNA motivates continued prioritization of both sample preparation and amplification methods designed for short fragments, although the latter will need to be balanced against reduced specificity accompanied by interrogating shorter nucleotide fragments. A sensitive molecular assay targeting urine cfDNA, rather than sputum, would considerably contribute to improving sample accessibility and diagnostic yield, and has the potential to advance the availability of rapid TB diagnostics across underserved patient populations. In addition, the combination of Q Sepharose DNA extraction and single-stranded library preparation techniques will be generally useful for other applications and contexts where the analysis of highly fragmented forms of DNA is necessary.

## Appendix Supplementary materials

Figure S1 Fragment length distribution of cfDNA mapped to the human genome in each sample. Image, application 1

Figure S2 Fragment length distribution of cfDNA mapped to the MTB genome in each sample. Sample 10 had a low number of reads that mapped to the MTB genome, but was confirmed to contain reads specific to MTB by metagenomic classification. Neither of the MTB-negative samples (sample 11 or sample 12, excluded here) contained reads specific to MTB by metagenomic classification. Image, application 2

Table S1 Demographic and clinical data, cfDNA concentration, and cfDNA fragment length for each participant Image, application 3

Table S2 Sequencing library metrics for each participant Image, application 4
