# Supplementary material for: Characterizing the molecular composition and diagnostic potential of Mycobacterium tuberculosis urinary cell-free DNA using next-generation sequencing
Source: Int J Infect Dis. 2021 Nov;112:330–7. doi: 10.1016/j.ijid.2021.09.042 (PMC8627387; doi:10.1016/j.ijid.2021.09.042)
Supplement: Supplementary file 3 — Table S1 Demographic and clinical data, cfDNA concentration, and cfDNA fragment length for each participant [file mmc3.pdf]

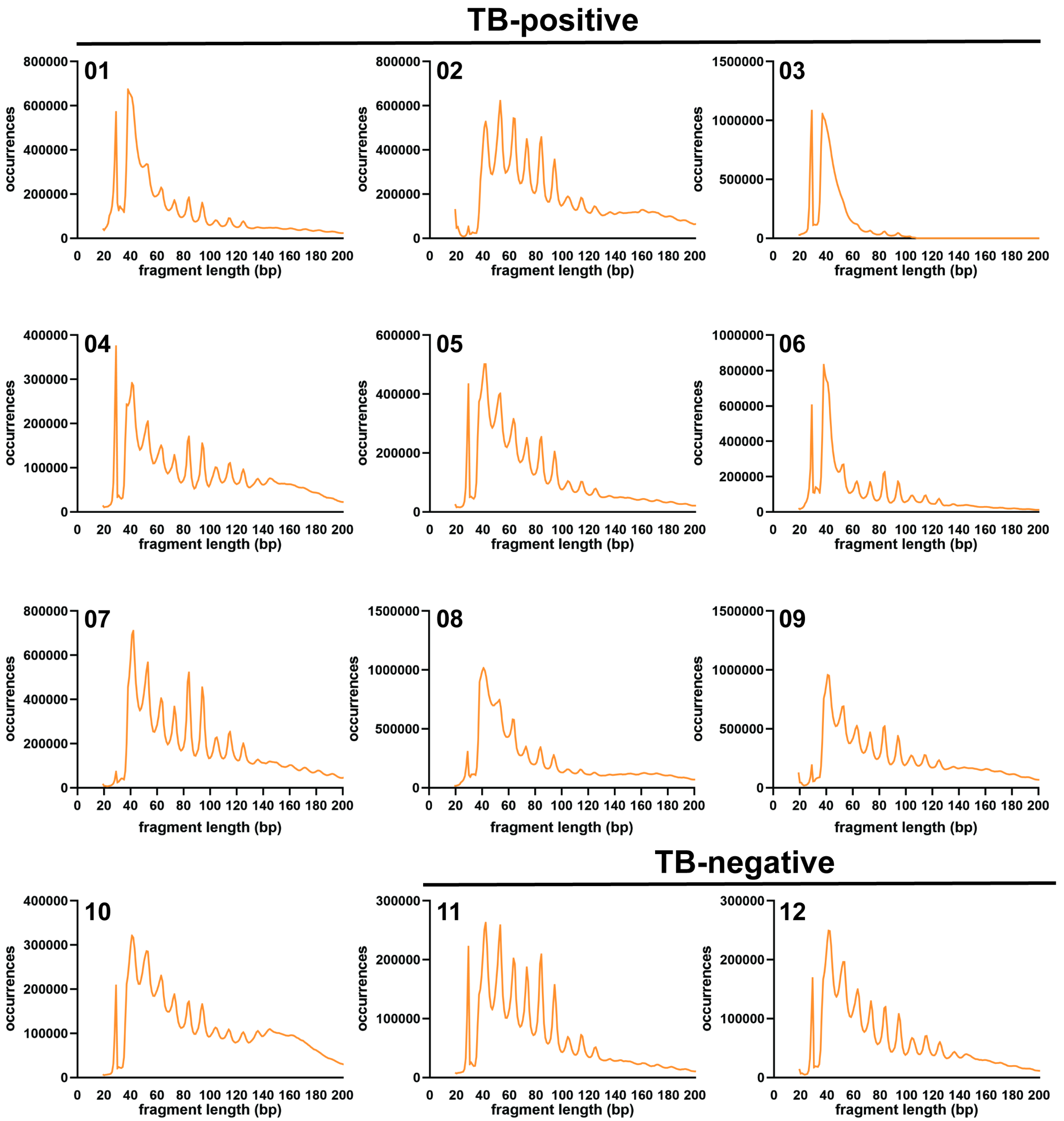

Supplemental Fig. S1: Fragment length distribution of cfDNA mapped to the human genome in each sample.
