# Supplementary material for: Characterizing the molecular composition and diagnostic potential of Mycobacterium tuberculosis urinary cell-free DNA using next-generation sequencing
Source: Int J Infect Dis. 2021 Nov;112:330–7. doi: 10.1016/j.ijid.2021.09.042 (PMC8627387; doi:10.1016/j.ijid.2021.09.042)
Supplement: Supplementary file 4 — Table S2 Sequencing library metrics for each participant [file mmc4.pdf]

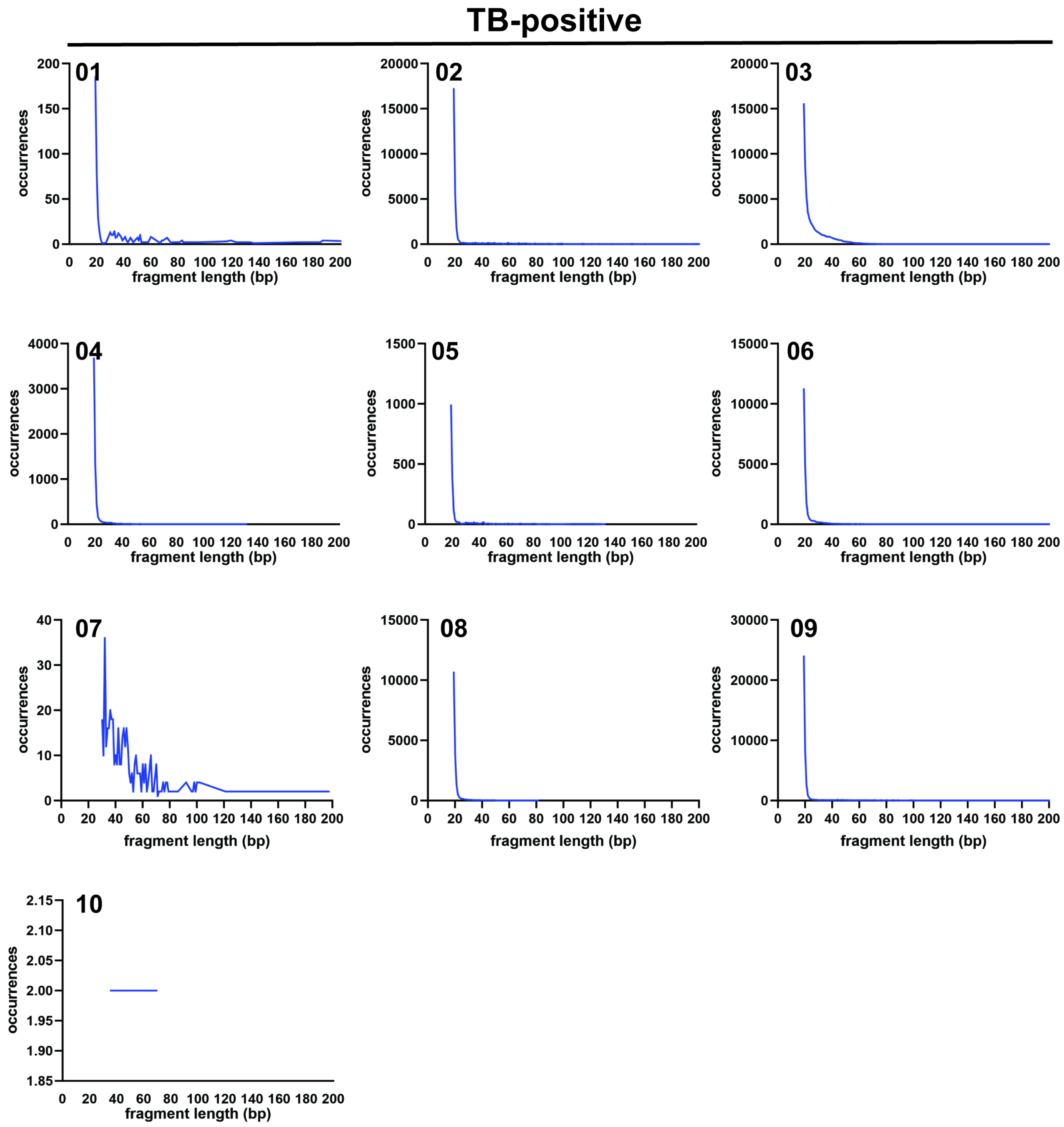

Supplemental Fig. S2: Fragment length distribution of cfDNA mapped to the TB genome in each sample. Sample 10 had a low number of reads that mapped to the TB genome, but was confirmed to contain reads specific to TB by metagenomic classification. Neither TB-negative sample (Sample 11 or Sample 12, excluded here) contained reads specific to TB by metagenomic classification.
